# Occupational Injuries and Illnesses among Paramedicine Clinicians: Analyses of US Department of Labor Data (2010 – 2020)

**DOI:** 10.1017/S1049023X23006118

**Published:** 2023-10

**Authors:** Brian J. Maguire, Ala’a Al Amiry, Barbara J. O’Neill

**Affiliations:** 1. Leidos Inc., Reston, Virginia USA; 2. Central Queensland University - School of Medical and Applied Sciences, Queensland, Australia; 3. College of Pharmacy and Health Sciences, Ajman University, Ajman, United Arab Emirates; 4. School of Nursing, University of Connecticut, Storrs, Connecticut USA

**Keywords:** Emergency Medical Services, epidemiology, illness, injury, occupational safety

## Abstract

**Objective:**

Paramedicine clinicians (PCs) in the United States (US) respond to 40 million calls for assistance every year. Their fatality rates are high and their rates of nonfatal injuries are higher than other emergency services personnel, and much higher than the average rate for all US workers. The objectives of this paper are to: describe current occupational injuries among PCs; determine changes in risks over time; and calculate differences in risks compared to other occupational groups.

**Methods:**

This retrospective open cohort study of nonfatal injuries among PCs used 2010 through 2020 data from the US Department of Labor (DOL), Bureau of Labor Statistics; some data were unavailable for some years. The rates and relative risks (RRs) of injuries were calculated and compared against those of registered nurses (RNs), fire fighters (FFs), and all US workers.

**Results:**

The annual average number of injuries was: 4,234 over-exertion and bodily reaction (eg, motion-related injuries); 3,935 sprains, strains, and tears; 2,000 back injuries; 580 transportation-related injuries; and over 400 violence-related injuries. In this cohort, women had an injury rate that was 50% higher than for men. In 2020, the overall rate of injuries among PCs was more than four-times higher, and the rate of back injuries more than seven-times higher than the national average for all US workers. The rate of violence-related injury was approximately six-times higher for PCs compared to all US workers, seven-times higher than the rate for FFs, and 60% higher than for RNs. The clinicians had a rate of transportation injuries that was 3.6-times higher than the national average for all workers and 2.3-times higher than for FFs. Their overall rate of cases varied between 290 per 10,000 workers in 2018 and 546 per 10,000 workers in 2022.

**Conclusions:**

Paramedicine clinicians are a critical component of the health, disaster, emergency services, and public health infrastructures, but they have risks that are different than other professionals.

This analysis provides greater insight into the injuries and risks for these clinicians. The findings reveal the critical need for support for Emergency Medical Services (EMS)-specific research to develop evidence-based risk-reduction interventions. These risk-reduction efforts will require an enhanced data system that accurately and reliably tracks and identifies injuries and illnesses among PCs.

## Introduction

Emergency Medical Services (EMS) professionals risk their health and safety every day. While caring for the 40 million people who call for their help every year in the United States (US),^1^ they often operate in patients’ workplaces and homes, or in dangerous environments such as multi-vehicle collisions, shootings, pandemics, and other disasters. The almost one million EMS professionals in the US^1–3^ include emergency medical technicians (EMTs) and paramedics. Although efforts to fully describe this workforce have been unsuccessful,^4^ estimates are that approximately one-quarter are employed while the majority are volunteers.^5^ The EMS professionals are herein referred to collectively as paramedicine clinicians (PCs). The PCs typically work in over 20,000 EMS agencies in the US.^3^ More recently, they are also becoming involved in community paramedicine roles including preventive health services for under-served populations.^6^

The first research to evaluate PCs’ risk of occupational fatality found that this group had a rate almost three-times higher than the national average; this is comparable to the rates for police and fire fighters (FFs).^7^ Transportation-related trauma was the leading cause of the PCs’ fatalities.^7,8^ A recent analysis found that 75% of occupational fatalities among PCs were transportation-related.^9^

The first study to compare PC rates of nonfatal injuries to other occupational groups found that PCs had an injury rate seven-times higher than the national average.^10^ Later studies determined that each year, over 400 clinicians sustained serious assault-related injuries^11^ and an approximately equal number sustained serious transportation-related injuries.^12^ Findings of high risks for PCs are not unique to the US; studies in Australia found that paramedics have higher risks of violence-related injury and higher injury and fatality rates than any other occupational group.^13,14^

Furthermore, PCs typically do not have immediate access to tests to determine whether the patients they encounter have a communicable disease. They transport their patients in confined and enclosed vehicles that often have poor ventilation, rendering these vehicles prone to being colonized by multiple microorganisms.^15,16^ As a result, the PCs have high rates of infectious diseases, including coronavirus disease 2019 caused by the SARS-CoV-2 virus (COVID-19) and Methicillin-resistant Staphylococcus aureus.^17–20^

Current information is needed to develop targeted evidence-based risk-reduction interventions for the PCs. Reducing occupational risks for this group will: enhance the health of the workforce, increase communities’ access to care, and will ultimately help these clinicians to provide the best possible care to the millions of people who call for their help every year.

The research objectives for this study are to: determine the rates of injuries among PCs; determine the types of occupational risks among PCs; describe how the risks have changed over time; and calculate the differences in their risks compared to other workers in the US.

## Methods

### Study Design

This is a retrospective open cohort study of nonfatal occupational injuries among PCs in the US.

### Data Sources/Measurement

The US Department of Labor (DOL), Bureau of Labor Statistics (Washington, DC USA) provides online tools to access its occupational injury, illnesses, and fatalities data.^21^ The DOL has collected occupational injury and fatality data since before World War I; it has published industry data since 1972 and occupation data since 1992. Their data and methods have been previously reported.^11^ The DOL provides nonfatal injury and illness data for all occupational injuries and for nonfatal cases involving days away from work.^22^ Cases are included if they result in any of the following: loss of consciousness; days away from work; restricted work activity or job transfer; or medical treatment beyond first aid.^23^ For some analyses, only cases that resulted in at least one day of lost work time were used. The data exclude work-related fatalities and nonfatal work injuries and illnesses to the self-employed, to volunteers, and to federal government workers.^24^ The DOL data include US workers over 16 years of age and working in the 50 US States, Guam, Puerto Rico, and the US Virgin Islands.^25^ The DOL data are the “only source of national-level data on nonfatal injuries and illnesses that spans the private sector and state and local government.”^26^ The DOL continually works to improve the accuracy and reliability of the data.^26^

As an example, data were extracted from the DOL site^27^ by going to the inquiry page and then selecting the needed data. Multiple searches using the same search criteria consistently returned identical results. Data on the number of individuals by sex and age group were accessed through the DOL’s labor force search engine.^28^ Note that although illnesses that meet the above criteria are included in the total number of cases, the available data identify no individual illnesses.

### Setting and Population

The population for this study includes those individuals classified by DOL as “EMTs and paramedics” for the period of 2010 through 2020. Herein they are referred to collectively as PCs. Since they are reasonably similar professions, FFs and registered nurses (RNs) were selected for comparison. The DOL category of RN includes Clinical Nurse Specialists but excludes Nurse Practitioners, Nurse Anesthetists, and Nurse Midwives.^29^

### Bias

This is an examination of secondary data previously collected by the DOL. Participants were unable to choose to be included or excluded. Selection bias is a common concern for retrospective database analyses.^30^ Anecdotal reports indicate that PCs may be less inclined to report an injury or illnesses compared to other workers for various reasons, including missing time with another employer or being treated by a colleague or self-treated. The PCs may have very high rates of occupational illnesses, but if the illness manifests days (or longer) after exposure, the illness might not be categorized as occupational. The data collected are, by DOL definition, only a subsection of the total number of occupational injuries and illnesses and so may not be reflective of actual risks. As noted in the Limitations section, it is likely that many cases that should have been included in this analysis were instead categorized as happening to other occupational groups, including FFs. Injuries to EMTs and paramedics who are full-time FFs are categorized as FF injury. It is unknown if injuries to PCs who are employed by fire departments, but who are not FFs, are categorized as FF.

### Quantitative Variables

The DOL provides variables that include the sex and age of injured workers and counts of injuries by factors such as nature of injury, source of injury, event, and body part injured. Not all variables were available for all years. Person-years is the total number of personnel each year summed for the time period. Data on potential confounders such as previous injury, work hours, drug and alcohol use, number of full and part-time jobs, and fatigue were not available.

### Statistics and Data Analyses

The rates and relative risks (RRs) of injuries among PCs were compared against the rates of FFs, RNs, and all US workers using a confidence interval (CI) of 95% for the RR. Injury rates were calculated using the formula: Rate = (cases/population) *10,000. For an examination of how risks for PCs compared to risks for FF and RNs, data on cases and rates for PCs, FFs, RNs, and the US population were downloaded from the DOL search engine site.^31^ Population totals were derived by solving for population using the formula: rate = (cases/population) *10,000. The RR was calculated using the formula: RR = rate for Group 1 (PCs)/rate for Group 2. The CIs were computed using the formula: Ln(RRhat)+/-z√(((n1-x1)/x1)/n1)+(((n2-x2)/x2)/n2),^32^ where n1 was the number of cases among PCs, x1 was the PC population, n2 was the number of fatalities in the comparison group, x2 the comparison group population, and z = 1.96 (for the 95% CI); finally, the antilogs of the lower and upper limits were computed. Any RRs with CIs that did not include “1” were considered statistically significant.

Data were entered into a Microsoft Excel spreadsheet and analyses were done using Microsoft Excel 365 (Microsoft Corp.; Redmond, Washington USA).

The paper follows the Strengthening the Reporting of Observational Studies in Epidemiology (STROBE) Statement guidelines.^33^

### Ethics

This study was approved by the Research Ethics Committee of Ajman University, Ajman, United Arab Emirates (Reference number: D-H-F-11-Nov).

## Results

### Population Demographics

**Figure 1.**
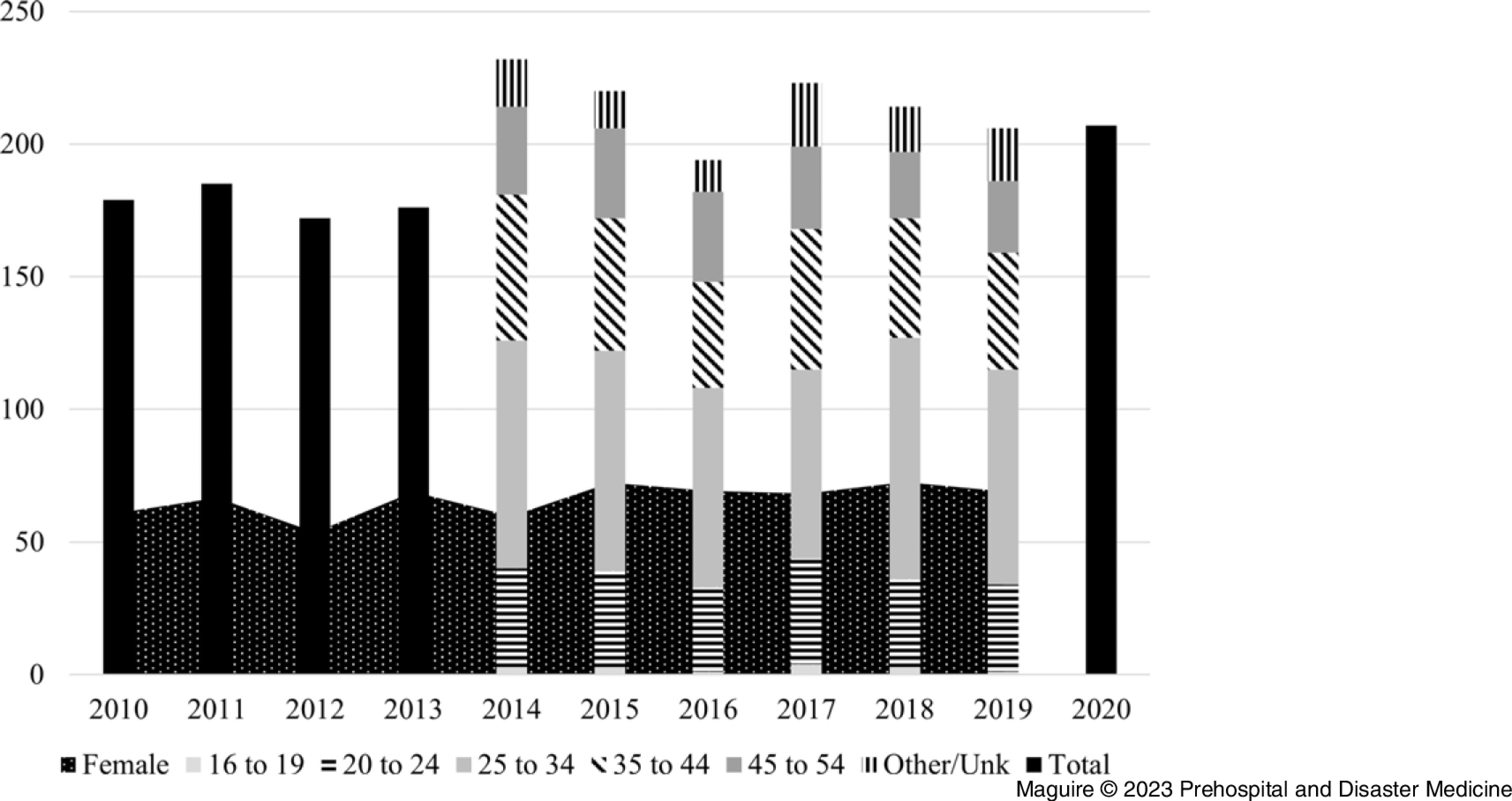
Number (in thousands) of Employed Paramedicine Clinicians per Year for 2010 through 2020, with Number by Age Group for 2014 through 2019 and Number of Females for 2010 through 2019 (n = 2,208,000 person-years).

Figure 1 shows that the number of employed PCs per year from 2010 through 2020 varied between a low of 172,000 in 2012 to a high of 232,000 in 2014 for a total of 2,208,000 person-years (average: 200,727; standard deviation [SD] = 20,712). A breakdown by age group was only available for the years of 2014 through 2019. For those years, the average proportion by group was one percent for 16 to 19, 16% for 20 to 24, 38% for 25 to 34, 22% for 35 to 44, and 14% for 45 to 54 year-old categories; in eight percent of cases, the age was other or unknown. The number of women was available from 2010 through 2019; the average number of women across those years was 66,157 (approximately 33% of the population).

### Outcome Data

From 2011 through 2020, the total number of injury and illness cases resulting in lost work time among PCs was 77,280. The total number of cases per year varied from a low of 6,640 in 2016 to a high of 11,270 in 2020 (average: 7,728; SD = 1,326).

Data on the number of personnel and the number of cases by sex and age were available from the DOL only for 2014 through 2019. Table 1 shows that from 2014 through 2019, the overall rate of cases per 10,000 persons was 427.0 for women, 288.7 for men, and 333.7 for all PCs. The RR for women compared to men was 1.5 (CI = 1.35, 1.63). The RR by age compared to those 25 to 34 years old increased for those 35 to 44 (RR = 1.3; CI = 1.18, 1.45) and for those 45 to 54 (RR = 1.4; CI = 1.30, 1.57).

**Table 1.** Nonfatal Occupational Injury Cases Involving at least One Day Away from Work for Paramedicine Clinicians by Sex and Age

	Person Years	Cases	Rate	RR	CI Low	CI Upper
Women	411,288	17,560	427.0	1.5	1.35	1.63
Men	877,712	25,340	288.7			
Other/Unknown		120				
20 – 24	211,000	6,360	301.4	1.0	0.93	1.16
25 – 34	487,000	14,150	290.6	1.0		
35 – 44	287,000	10,900	379.8	1.3	1.18	1.45
45 – 54	184,000	7,630	414.7	1.4	1.30	1.57
Other/Unknown	120,000	3,980	331.7			
Total	1,289,000	43,020	333.7			

Note: Total person years, total cases, rate per 10,000 workers, relative risk (RR), and the 95% confidence interval (CI) of the RR. (Cumulative data for 2014 to 2019; n = 1,289,000 person/years).

Abbreviations: RR, relative risk; CI, confidence interval.

### Main Results

*Case Types*—Data available from 2011 through 2020 showed that PCs had an annual average of: 3,935 sprains, strains, and tears injuries; 4,234 over-exertion and bodily reaction; 2,435 back injuries; 1,076 falls, slips, and trips; 580 transportation-related injuries; 426 violence-related injuries; and, on average each year, 101 PCs suffered “multiple traumatic injuries.” The source of the injury for approximately one-third of the cases was a “health care patient.” Over one-half of all cases were classified as “musculoskeletal disorders;” the rate of musculoskeletal injuries for PCs in 2020 (154.9) was six-times higher than the national average for all workers (26.9).

**Figure 2.**
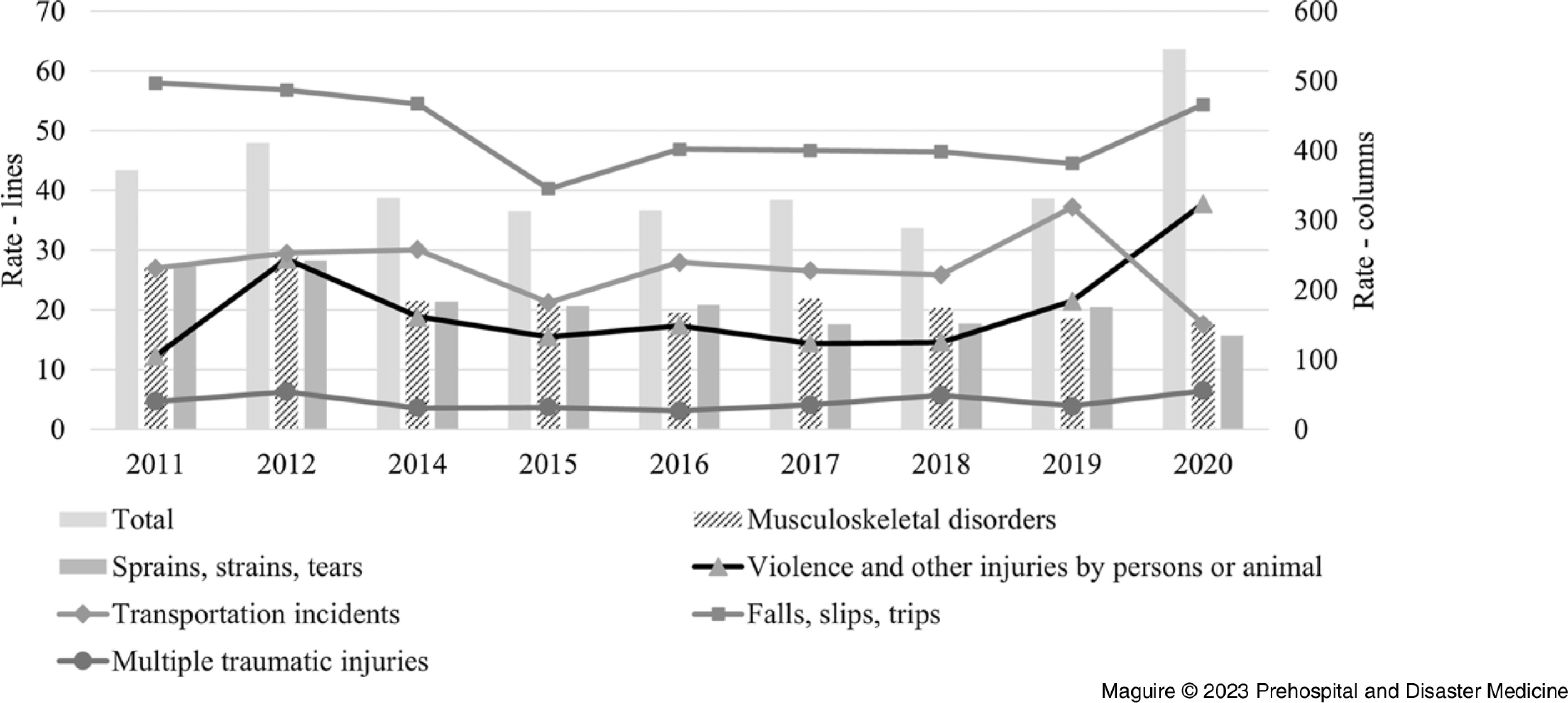
Incidence Rates per 10,000 Full-Time Workers for Nonfatal Occupational Injuries Involving Days Away from Work, by Total and Case Type, per Year, among Paramedicine Clinicians for 2011 through 2020 (n = 2,029,000 person-years). Note: Higher rates illustrated by bars corresponding to the right axis and lower rates illustrated by lines corresponding to the left axis.

Figure 2 shows the rates per year for: musculoskeletal disorders; sprains, strains, and tears; violence and other injuries by persons or animal; transportation incidents; falls, slips, and trips; and multiple traumatic injuries. The trends indicated a decrease in rates for musculoskeletal disorders and for sprains, strains, and tears, but an increase in the rate of violence in 2020.

*Lost Work Time*—Almost one-quarter (23.9%) of the 77,280 cases resulted in 31 or more days of lost work time. For 11% of the cases, the PCs lost one day of work; 10% lost two days; 18% of the cases resulted in three to five days of lost work time; 16% resulted in six to ten days; 14% had 11 to 20 lost days; and seven percent resulted in 21 to 30 days of lost work time. From 2011 through 2020, the average annual median days away from work for PCs was nine, compared to 12 for all US workers in 2020.

*Comparison to Other Workers*—Data on cases and rates were available from the DOL for all workers in the US, for RNs, FFs, and for all PCs, for the year 2020. Table 2 shows the rates of cases with at least one day of lost work time, by categories for the four groups, as well as the RRs and 95% CIs for PCs compared to the other three groups for 2020.

**Table 2.** Population, Total Cases, Incidence Rates of Nonfatal Occupational Injuries and Illnesses Involving at least One Day Away from Work by Selected Characteristics for All US Workers, Fire Fighters (FF), Registered Nurses (RN), and Paramedicine Clinicians (PC) for 2020

	US	FF	RN	PC
Population	111,993,711	331,911	2,340,254	206,562
Cases				
Total Cases	1,424,560	17,890	90,170	11,270
Violence	75,950	180	5,390	780
Transportation Incidents	54,750	250	550	360
Falls, Slips, Trips	256,830	2,460	6,690	1,120
Back Injuries	152,740	2,160	7,050	2,050
Multiple Traumatic Injuries	28,910	90	730	130
Rates				
Total Cases	127.2	539.0	385.3	545.6
Violence	6.8	5.4	23.0	37.8
Transportation Incidents	4.9	7.5	2.4	17.4
Falls, Slips, Trips	22.9	74.1	28.6	54.2
Back Injuries	13.6	65.1	30.1	99.2
Multiple Traumatic Injuries	2.6	2.7	3.1	6.3
RR for PC				
Total Cases	4.29	1.01	1.42	
Violence	5.57	6.96	1.64	
Transportation Incidents	3.57	2.31	7.42	
Falls, Slips, Trips	2.36	0.73	1.90	
Back Injuries	7.28	1.53	3.29	
Multiple Traumatic Injuries	2.44	2.32	2.02	
95% CI Low				
Total Cases	4.21	0.99	1.39	
Violence	5.19	5.92	1.52	
Transportation Incidents	3.21	1.97	6.49	
Falls, Slips, Trips	2.23	0.68	1.78	
Back Injuries	6.97	1.44	3.14	
Multiple Traumatic Injuries	2.05	1.77	1.67	
95% CI High				
Total Cases	4.37	1.04	1.44	
Violence	5.97	8.19	1.77	
Transportation Incidents	3.95	2.72	8.47	
Falls, Slips, Trips	2.51	0.78	2.02	
Back Injuries	7.60	1.62	3.46	
Multiple Traumatic Injuries	2.90	3.04	2.43	

Note: Includes rates per 10,000 workers with relative risk (RR) for clinicians compared to the other groups, and the 95% confidence interval (CI) of the RR (n = 206,562 PCs).

Abbreviations: US, United States; FF, fire fighters; RN, registered nurse; PC, paramedicine clinician; RR, relative risk, CI, confidence interval.

Compared to all workers in the US, the RRs for PCs in 2020 were: 5.6-times higher for violence; 3.6-times higher for transportation incidents; 2.4-times higher for falls, slips, and trips; 7.3-times higher for back injuries; and 2.4-times higher for multiple traumatic injuries. The overall injury rate for PCs was more than four-times higher than the rate for all US workers.

Compared to FFs, the rate for PCs was seven-times higher for violence-related injuries, more than twice as high for both transportation incidents and for multiple traumatic injuries, and over 50% higher for back injuries. The PCs had a lower rate than FFs for falls, slips, and trips, and overall, the rate of injuries was approximately one percent higher for PCs, but that RR was not statistically significant.

Compared to RNs, the RR for PCs was approximately seven-times higher for transportation incidents, three-times higher for back injuries, nearly twice as high for both falls, slips, and trips and multiple traumatic injuries, and approximately 60% higher for violence-related injuries. Overall, PCs had an injury rate almost 40% higher than RNs.

## Discussion

### Key Results

Paramedicine clinicians play a key role in public health and public safety, but they face multiple occupational health risks. This research documents that their injury rate is more than four-times higher than the national average for all workers in the US. On average, they suffer 7,728 injuries each year that are serious enough to cause at least one day away from work. Findings in this research highlight not only who is being injured, but also the causative mechanisms of their injuries, indicating areas where evidence-based interventions and more precise injury data are needed.

### Sex

Women make up approximately 33% of the PC population and have an injury rate that is 50% higher than for men. Other research has documented that women in this profession have higher rates of injuries compared to men.^8,10^ This is in contrast to the overall population in the US where the injury rate is lower for women.^34^ These findings suggest an urgent need for sex-specific injury research for PCs.

### Back and Musculoskeletal Injuries

The data show that PCs are at high risk of back injury; this is likely due to the strenuous nature of their work. The PCs rate now is more than seven-times higher than for all US workers. In 2007, their back injury rate was just five-times higher than the national average.^8^ This may indicate a continuous increase in rates related to the lack of effective risk-reduction interventions, despite anecdotal reports of increasing usage of devices meant to reduce back injuries.

Potentially one of the most hazardous lifting activities for PCs is lifting the stretcher into the ambulance; Prairie, et al note that this activity creates high risks for back injuries and that efforts to reduce the risk of injury while loading a stretcher should include evaluations of equipment, training, workers, and work organization.^35^

The PCs have a rate of musculoskeletal disorders (eg, soft-tissue injuries caused by repetitive motion) that is six-times higher than the national average for all workers. Musculoskeletal disorders are most likely the result of the nature of their prehospital duties of lifting and moving heavy objects and patients. Among Australian paramedics, 44% of all injuries were classified as muscular stress while lifting, carrying, or putting down objects.^13^

A variety of personal protection technology (PPT) options are available to reduce back injuries and other musculoskeletal injuries and disorders, yet more specific research is still needed to test individual PPT options for their injury prevention effectiveness among PCs. Beyond PPT improvements, another intervention to evaluate is exercise. It is possible that many of the musculoskeletal disorders, back injuries, and trunk injuries could be mitigated by regular strength-training exercises. Research has found that exercise provides many positive health benefits for workers.^36^ Future research should determine the correlation between physical fitness and these specific occupational injuries as a step toward developing and testing risk-reduction interventions.

Research focused on the relative value of ergonomic interventions found a significant decrease in injuries after implementing the use of powered stretchers.^37^ Such research should be further explored, including for devices such as descent control systems.

### Violence

The violence-related injury rate for PCs was 5.2 in 2007^8^ and 37.8 in 2020. One possible explanation for the higher number of cases in 2020 could be that the COVID-19 pandemic and its significant impact on dispatch centers and EMS personnel^38^ may have contributed to community stresses that led to a high rate of violence. The source of the injury for approximately one-third of all violence cases among PCs was a “health care patient.” Other research also found that many cases among PCs were caused by patients.^10,39^

The rate of violence-related injury is approximately six-times higher for PCs compared to all US workers, seven-times higher than the rate for FFs, and 64% higher than the rate for RNs. An analysis of data from one large fire department that employed both FFs and PCs found that the rate of assault-related injuries among the PCs was 40-times higher than the rate for the FFs.^40^ Maguire and O’Neill found that PC women had a disproportionately greater risk of violence-related injuries.^11^ As with health care in general,^41^ studies on violence prevention for PCs are urgently needed.

The PPT that would seem to be the most effective in reducing violence-related risks are bullet- and stab-proof vests. However, only approximately 10% of violence-related injuries among PCs were caused by a perpetrator with a weapon.^42^ A survey of 633 PCs who had been victims of violent attack found that not one indicated that they thought a vest would have helped.^43^ Further, it is possible that these vests might increase occupational hazards for PCs.^44^ Additional research is needed to determine how PPT might reduce risks for PCs.

### Transportation

Paramedicine clinicians have a rate of transportation injuries that is almost four-times higher than the national average for all US workers, more than twice as high as for FFs, and seven-times higher than for RNs. This finding is similar to a 2005 report that showed a very high rate (34 cases per 100 workers per year) of transportation-related injuries for these personnel.^10^ Transportation-related fatalities occurred at a rate of 9.6 per 100,000 per year, approximately five-times higher than for all US workers and higher than the rates for police officers and FFs (6.1 and 5.7, respectively).^7^ Approximately 75% of fatalities among PCs are transportation-related.^9^ Although vehicle safety is part of training, the risk of transportation injuries among PCs remains high. Details of where these workers were injured are limited, namely if the incidents occurred outside the ambulance (eg, struck by another vehicle while caring for a patient on the road), inside the ambulance patient compartment, or inside the driver’s compartment in the ambulance. Transportation-related risks can be reduced,^45^ and behaviors such as wearing seat belts can substantially reduce risks.^46^ There are PPT that can reduce risks for these clinicians (eg, wearable and lift-assist devices),^47,48^ but evidence on their effectiveness for PCs is lacking. Future research should determine barriers to using the available PPT and determine the best PPT for each specific environment, as well as ways of reducing overall risks of transportation-related injuries for PCs.

### Factors Potentially Associated with Increased Risks

Injury risks for paramedics are significantly related to fatigue.^49^ Anecdotal information suggests that many PCs work multiple jobs. Fatigue may also produce a higher level of impairment than legal limits of alcohol consumption,^50,51^ and may be associated with increased risks of injury and of making medical errors.^52^

In addition to fatigue, a variety of factors including stress and high-risk alcohol and other drug use may be associated with increased risks of occupational injury for PCs.^53^ In addition to potentially increasing risks for PCs, these factors may also contribute to increasing risks for patients being treated by PCs, and for members of the public. Additional research is needed to investigate the association between such factors and occupational risks, as well as how any risks could be mitigated.

### Resource Gaps

On November 2, 2021, a Federal Register notice announced that the National Institute for Occupational Safety and Health (NIOSH; Washington, DC USA) was seeking input on the need to establish “centers of excellence to address research and practice needs in the area of personal protective technology (PPT).”^48^ A new National Highway Traffic Safety Administration (NHTSA; Washington, DC USA) goal of zero roadway fatalities^54^ is an indicator that a goal of zero PC transportation-related occupational fatalities is both reasonable and appropriate. The findings in this study support the need for an EMS center of excellence.

### Generalizability

Although the capabilities of EMS systems in the US have fallen behind their counterparts in other developed countries,^55^ it remains likely that many of the occupational risks seen in this study will be similar to the risks among PCs internationally.

## Limitations

The 261,300 PCs in the DOL database in 2020^56^ are approximately one-quarter of the almost one million PCs estimated to be in the US.^1,2^ Further, because some portion of PCs in the US are employees (or members) of fire departments, some unknown number of injury and illness cases among PCs are categorized as occurring to a FF, perhaps even if the PC was a non-FF employee of a fire department, or was a FF working in a strictly EMS role. That likely results in decreasing the rates for PCs and increasing the rates for FFs, thereby also decreasing the RR. In addition, an unknown number of PCs employed by police departments and health care agencies might also have their injury and illness cases classified as occurring to police officers or health care workers; this again would result in lower cases, rates, and RRs for PCs.

The length of service findings must be interpreted with caution because there are no data on the number of workers in each of the length of service categories.

Absent from the available data are any way to identify mental-health-related incidents affecting PCs as related to their work environment. Also absent from the data are any categories describing illnesses; illnesses are included in the total number of cases for the year, but there are no indications of how many of the cases are illnesses nor how many are specific illnesses such as COVID-19.

The PCs have very different call volumes based on factors such as working at an urban or rural agency. It is likely that the risks of injury are correlated with call volume. Call data were not available for this study, but future research should examine how various factors including call volume, call type, and work location are associated with overall and specific injury risks.

While providing useful insights, the findings in this research also highlight the inadequacies of the currently available data and support the need for a data system more specific to the needs of PCs.^57^ For example, although it is likely the result of COVID-19, the reasons that the rate of “exposure to harmful substances” for PCs went from 6.6 in 2007^8^ to 234.4 in 2020 is unknown. It is expected that risks for these professionals vary widely by various factors, work location, and even call type, yet none of those data are currently available. A goal for future research should be the creation of an EMS database that links agency level operations data with medical records and personnel data at the person level; such a database has been shown to be an effective resource for identifying and describing occupational risks.^58^ Such a database would also allow for analyses of a variety of potential confounders.

Reducing occupational risks for PCs requires: (1) occupation-specific research funding; (2) enhanced data systems that include agency-level and person-level operations, medical, and personnel data; (3) a re-evaluation of current EMS training curricula; (4) improved PPT; (5) studies of improving physical fitness; and (6) new training focused on increasing situational awareness and safety preparedness.

## Conclusion

From these analyses of DOL data, there is now greater insight into who is being injured and where evidence-based interventions, and more precise injury data, are needed to protect PCs in the future.

In the US, PCs respond to 40 million calls for help each year. While providing this critical care, these clinicians suffered from an injury rate of 545.6 per 10,000 persons in 2020; this is a rate more than four-times higher than the national average for all US workers.

The major types of occupational injuries included musculoskeletal disorders, back injuries, transportation-related injuries, and violence-related injuries.

The findings that PCs have a 2.3-times higher risk of injuries than FFs for transportation-related injuries, a seven-times higher rate than FFs for violence-related injuries, and a 60% higher injury rate than RNs demonstrate the urgent need for paramedicine-specific resources to reduce these risks. The data limitations, including the absence of data identifying mental-health-related incidents and illnesses, demonstrate the serious shortcomings of the currently available data.

The findings in this research support the critical need for paramedicine-specific occupational risk research.
